# Effect of Heating and Glycation on the Allergenicity of 2S Albumins (Ara h 2/6) from Peanut

**DOI:** 10.1371/journal.pone.0023998

**Published:** 2011-08-25

**Authors:** Yvonne M. Vissers, Fany Blanc, Per Stahl Skov, Phil E. Johnson, Neil M. Rigby, Laetitia Przybylski-Nicaise, Hervé Bernard, Jean-Michel Wal, Barbara Ballmer-Weber, Laurian Zuidmeer-Jongejan, Zsolt Szépfalusi, Janneke Ruinemans-Koerts, Ad P. H. Jansen, Huub F. J. Savelkoul, Harry J. Wichers, Alan R. Mackie, Clare E. N. Mills, Karine Adel-Patient

**Affiliations:** 1 Cell Biology and Immunology Group, Wageningen University and Research Centre, Wageningen, The Netherlands; 2 Food Chemistry Group, Wageningen University and Research Centre, Wageningen, The Netherlands; 3 Unité d'Immuno-Allergie Alimentaire UR496, Département Alimentation Humaine, INRA, Jouy-en-Josas, France; 4 RefLab ApS, Copenhagen, Denmark; 5 Institute of Food Research, Norwich Research Park, Colney, Norwich, United Kingdom; 6 Allergy Unit, Department of Dermatology, University Hospital Zurich, Zurich, Switzerland; 7 Laboratory of Allergy Research, Department of Experimental Immunology, Academic Medical Centre, University of Amsterdam, Amsterdam, The Netherlands; 8 Department of Pediatrics, Medical University of Vienna, Vienna, Austria; 9 Department of Clinical Chemistry and Haematology, Rijnstate Hospital, Arnhem, The Netherlands; 10 Department of Otorhinolaryngology, Radboud University Nijmegen Medical Centre, Nijmegen, The Netherlands; 11 Food and Biobased Research, Wageningen University and Research Centre, Wageningen, The Netherlands; Ulm University, Germany

## Abstract

**Background:**

Peanut allergy is one of the most common and severe food allergies, and processing is known to influence the allergenicity of peanut proteins. We aimed to establish the effect of heating and glycation on the IgE-binding properties and biological activity of 2S albumins (Ara h 2/6) from peanut.

**Methodology/Principal Findings:**

Native Ara h 2/6 was purified from raw peanuts and heated in solution (15 min, 110°C) in the presence or absence of glucose. Ara h 2 and 6 were also purified from roasted peanut. Using PBMC and sera from peanut-allergic patients, the cellular proliferative potency and IgE reactivity (reverse EAST inhibition) and functionality (basophil degranulation capacity) of allergens were assessed. Heating Ara h 2/6 at 110°C resulted in extensive denaturation, hydrolysis and aggregation of the protein, whilst Ara h 2 and 6 isolated from roasted peanut retained its native conformation. Allergen stimulation of PBMC induced proliferation and Th2 cytokine secretion which was unaffected by thermal processing. Conversely, IgE reactivity and functionality of Ara h 2/6 was decreased by heating. Whilst heating-glycation further reduced the IgE binding capacity of the proteins, it moderated their loss of histamine releasing capacity. Ara h 2 and 6 purified from roasted peanut demonstrated the same IgE reactivity as unheated, native Ara h 2/6.

**Conclusions/Significance:**

Although no effect of processing on T-cell reactivity was observed, heat induced denaturation reduced the IgE reactivity and subsequent functionality of Ara h 2/6. Conversely, Ara h 2 and 6 purified from roasted peanut retained the structure and IgE reactivity/functionality of the native protein which may explain the allergenic potency of this protein. Through detailed molecular study and allergenicity assessment approaches, this work then gives new insights into the effect of thermal processing on structure/allergenicity of peanut proteins.

## Introduction

 Peanut allergy is relatively common in the USA and certain European countries with the prevalence of sensitization being estimated as 2% and clinical peanut allergy as 1.2% of 3–4 years old children in the UK [1]. Whilst the incidence appears to be stabilising in the UK [1], it is still rising in the USA [2]. The peanut 2S albumins Ara h 2 and Ara h 6 together with a third low abundance 2S albumin, Ara h 7 have been identified as major peanut allergens [3], [4], [5]. Ara h 2 and 6 comprise several isoforms of Mr 17 kDa and 15 kDa, respectively [6], [7], [8]. Produced as a single chain precursor they are proteolytically processed in peanut seeds into two subunits linked by intramolecular disulphide bonds [6], [9]. Ara h 2, 6 and 7 are all members of the prolamin superfamily and share a characteristic cysteine skeleton with at least 8 conserved cysteine residues [9] and a three-dimensional structure comprising 5 α-helices arranged in a right-handed super helix. It appears this scaffold is stable to thermal processing and proteolysis [7], [10], [11].

Thermal processing of proteins can lead to alterations in their structure that can result in changes in their immunoreactivity/allergenicity. Typically, loss of tertiary structure is followed by reversible unfolding, while loss of secondary structure (70–80°C) leads to the formation of new intra/intermolecular interactions, rearrangements of disulfide bonds (80–90°C), and formation of aggregates (90–100°C) [12]. Heating in the presence of sugars found in the foods also leads to modification by the Maillard reaction (non-enzymatic browning). Free primary amino groups are attacked by carbonyl compounds during the Maillard reaction, leading to the formation of stable advanced glycation end products (AGE). Several studies have been performed to assess the IgE-binding capacity of purified allergens modified *in vitro* by heating and/or by Maillard reactions. In some cases, glycation of allergens enhanced their IgE binding capacity [13] or their T-cell immunogenicity [14], [15] whereas in other studies, glycation had no effect or caused even decreased IgE-binding capacity [16], [17]. Heating for 90 min at 100°C of recombinant refolded Ara h 2 led to a slight increase in its IgE binding capacity, which was further enhanced in the presence of glucose, maltose or ribose [18]. Heating native Ara h 2 for several days at 55°C in the presence of different sugars increased its IgE binding capacity compared to protein heated without sugar, which was related to the formation of AGE products [19]. Ara h 2 extracted from heat-processed peanut, such as roasting (140°C) was also found to enhance its IgE-binding capacity [20].

Although IgE binding capacities of modified allergens have been studied, sometimes with conflicting results, few data are available on the impact of heating on the protein structure and on the resultant biological activity of modified allergens compared to unmodified ones. In order to give new insights into the effect of thermal processing on structure/allergenicity of peanut proteins, we then purified and produced well-characterized native, heated and glycated Ara h 2/6, as well as corresponding protein from roasted peanut. Using a large panel of sera and peripheral blood mononuclear cells (PBMC) from well-characterized peanut-allergic patients recruited in different European countries, we then investigated the effect of thermal modifications on IgE reactivity of Ara h 2/6, but also on its biological activity, i.e. basophil activation, T-cell induced proliferation and cytokine production capacities.

## Results

### Effect of thermal processing on Ara h2/6 structure

**Figure 1:**
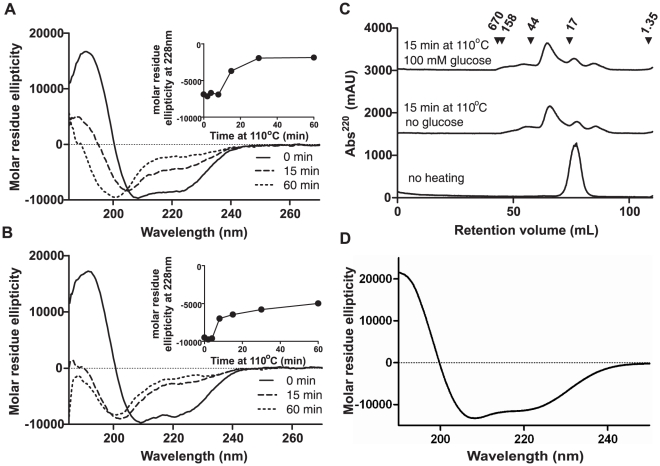
Effect of heating on secondary structure and oligomeric state of Ara h 2/6. Far-UV CD spectra of Ara h 2/6 heated alone (**A**, H-Ara h 2/6) or in the presence of 100 mM glucose (**B**, G-Ara h 2/6) before heating (N-Ara h 2/6, —) and for heated-cooled protein after heating to 110°C for 15 min (−−−) and 60 min (---). Inset graphs show change in molar residue ellipticity at 228 nm with heating time. Size exclusion chromatography profiles (**C**) are shown of the Ara h 2/6 before and after heating for 15 min at 110°C in the presence or absence of glucose. Retention volumes of molecular weight standards (size indicated in kDa) are shown by arrow heads. **D**. Far-UV CD spectra of Ara h 6 purified from roasted peanut.

Native Ara h 2/6 (N-Ara h 2/6) gave a far-UV CD spectrum typical of an α-helix rich protein (Figs 1A and 1B) consistent with previous studies [21] and was monomeric with a Mr of ∼16 kDa (Fig. 1C) consistent with a mixed preparation of Ara h 2/6 [7]. After heating at 110°C for 5–10 min in the absence [H-Ara h 2/6, Fig. 1A] or presence [G-Ara h 2/6, Fig. 1B] of glucose secondary structure shifted toward an unordered state which dominated after 15 min heating, with a loss of both the maximum at 198 nm and the minima at 209 and 222 nm. Heating in the presence or absence of glucose also affected the aggregation state of the protein, with only ∼20% of the protein remaining in its monomeric state (Fig. 1C). A broad peak eluting with a Mr ∼28–30 kDa of aggregated protein, together with a smear of material of Mr 44–150 kDa corresponding to larger oligomers was observed. In addition, thermal treatment resulted in hydrolysis of the Ara h 2/6 to yield fragments of smaller size than the parent protein. Far-UV CD spectra obtained for Ara h 2 (data not shown) and Ara h 6 (Figure 1D) purified from roasted peanut were comparable and demonstrated the same α-helix rich protein structure as N-Ara h 2/6. Non-denaturating electrophoresis showed the proteins were mainly monomeric (data not shown).

In order to study the effect of heat-induced structural changes on allergenicity of 2S albumins, N-Ara h 2/6 was then further heated for 15 minutes at 110°C in the presence or absence of glucose.

### Effect of thermal processing on PBMC induced proliferation and cytokine production capacity

**Figure 2:**
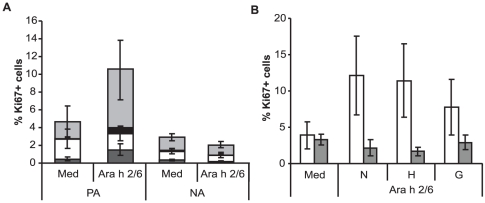
Effect of thermal treatment on subpopulation of proliferating cells. **A.** Fresh PBMC were left unstimulated (Med) or stimulated with N-Ara h 2/6 for 7 days. After harvesting, the cells were incubated with anti-CD4/CD25 antibody and afterwards stained for an anti-Ki-67 PE antibody. Data are mean percentage of Ki-67 positive cells ± SEM values for three (#65, #66 and #70) of the 12 PA subjects and seven NA controls. The percentage Ki-67+ cells was divided in the following subpopulations of cells: CD4-CD25- (dark grey bars), CD4+CD25- (white bars), CD4-CD25+ (black bars) and CD4+CD25+ (light grey) cells. **B.** Fresh PBMC were left unstimulated (Med) or stimulated with N-, H- or G-Ara h 2/6 for 7 days. Data are mean percentage of Ki-67 positive cells ± SEM values for three peanut-allergic patients (#65, #66 and #70) (white bars) and seven non-allergic controls (grey bars).

**Figure 3:**
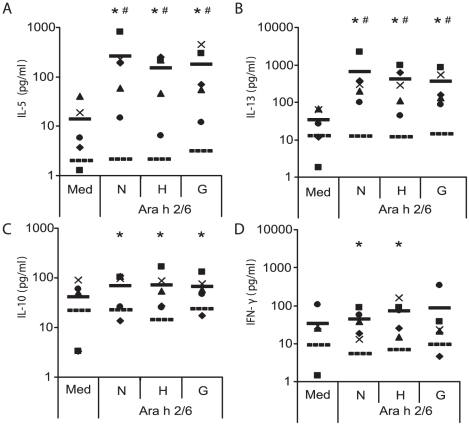
Effect of thermal treatment on cytokine induction capacity. Fresh PBMC were left unstimulated (Med) or stimulated with N-, H- or G-Ara h 2/6 and production of IL-5 (**A**), IL-13 (**B**), IL-10 (**C**) and IFN-γ (**D**) was measured after 7 days of culture. Symbols represent peanut-allergic patients #65 (▪), #66 (♦), #70 (▴), #67 (•) and #73 (×). Horizontal bars represent the mean of the five PA responder subjects (solid) and the mean of 7 NA controls (dotted). Asterisks indicate statistically significant differences between the PA and the NA group and # indicate statistically significant differences between the medium and allergen-stimulated cultures for the PA group (*, #: *P*<0.05). No differences between the medium and allergen-stimulated cultures were observed for the NA group.

No difference was observed between PBMC from PA or NA patients regarding differentiation markers, viability and cytokine secretion potency at the time of sampling and/or after polyclonal activation using αCD3/αCD28 antibodies (data not shown). The effect of thermal treatment on the capacity of Ara h 2/6 to stimulate T-cell proliferation and cytokine production by PBMC of peanut allergic patients was determined by culturing cells for 7 days in the presence or absence of native and thermally-modified Ara h2/6. Stimulation with Ara h 2/6 did not induce significant proliferation in the NA controls (Fig. 2A) and only three of the 12 PA subjects showed detectable numbers of Ki-67+ proliferating cells (10.5±4.8%) in the Ara h 2/6 stimulated cultures compared with medium alone (4.7±2.7). The CD4+CD25+ subset was the largest cell population present in the corresponding proliferating PBMC. Thermal treatment of N-Ara h 2/6 had no effect on its capacity to induce PBMC proliferation in the three subjects (Fig. 2B). PBMC cultures from NA subjects stimulated with the native and modified Ara h 2/6 did not show any cytokine induction for all five tested cytokines (Fig. 3 and data not shown). Conversely, IL-5 and IL-13 production was significantly and equally enhanced in PBMC cultures from 5 out of 12 PA subjects following stimulation with N-, H- or G-Ara h 2/6 (Figs 3A and 3B). Enhanced production of IL-10 and IFN-γ was observed in stimulated PBMC of PA subjects compared to NA subjects but was not significant for all stimuli tested (Figs 3C and 3D). IL-17 production was below 20 pg/ml for all tested conditions (data not shown).

### Effect of thermal processing on the IgE binding capacity

**Figure 4:**
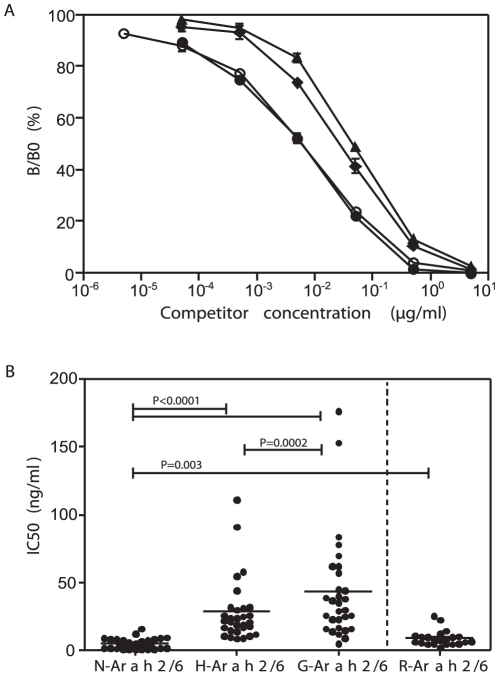
Effect of thermal treatment on the IgE binding capacity of Ara h 2/6. **A.** IgE capture inhibition curves obtained for sera #05-0209 with N-Ara h 2/6 (•), H-Ara h 2/6 (⧫) or G-Ara h 2/6 (▴). IgE binding capacities of native and heated processed Ara h 2/6 was assessed by competitive assays in the IgE capture format. Inhibition was performed into microtiter plates coated with anti-IgE, and previously incubated with allergic patient sera at convenient dilution. Competition was then conducted by adding increasing concentrations of competitors at the same time as biotinylated N-Ara h 2/6. Competition obtained with Ara h 2/6 purified from roasted peanut is shown as a clear circle. **B**. Analysis of IC50 (ng/ml) values obtained using an IgE capture inhibition assay with native and heat processed Ara h 2/6 as competitors using 30 individual sera from peanut-allergic patients. Increase in IC50 value corresponds to a decrease in IgE-binding capacity. R-Ara h 2/6: mix of Ara h 2 and Ara h 6 purified from roasted peanut. Bars indicate a significant difference between the 2 corresponding treatments (*P*<0.05, non-parametric Wilcoxon signed rank test).

Screening of the serum panel (n = 34, Table 1) showed variable levels of IgE specific to raw peanut extract and N-Ara h 2/6. Four sera with low specific IgE were excluded from subsequent analysis (sera #69, #76, #80 and #82). Effect of thermal treatment on the IgE binding capacity of Ara h 2/6 was then assessed using reverse inhibition. A typical inhibition curve for the reverse EAST assay obtained with serum #05-0209 is shown in Figure 4A and demonstrated that the thermal treatment of N-Ara h 2/6 at 110°C reduced its IgE binding capacity. Ara h 2/6 from roasted peanut (R-Ara h 2/6) had a similar albeit slightly reduced immunoreactivity to N-Ara h 2/6 from raw peanut. IC50 values calculated for the entire serum panel (n = 30) are consistent with this and show that extreme thermal processing did reduce the IgE binding capacity of Ara h 2/6 in a significant manner (Fig. 4B). The IC50 values for all tested sera increased 1.2 to 44-fold (mean  =  9.6, n = 30) for H-Ara h 2/6 compared to N-Ara h 2/6 (Fig. 4 B). For 25 out the 30 tested patients, IC50 values for G-Ara h 2/6 were equivalent or 3.4-fold higher than those of H-Ara h 2/6 (mean  =  1.7). For the last 5 patients, sampled in various countries, IC50 values were 1.2 to 1.7-fold lower for G-Ara h 2/6 compared to H-Ara h 2/6 (mean 1.4). These data show that for most patients glycation reduced the IgE reactivity of Ara h 2/6 still further compared with thermal treatment alone, and is consistent with the fact that IC50 values for G-Ara h 2/6 were 1.3 to 73-fold higher than that of N-Ara h 2/6 (mean  =  14.5, n = 30). Ara h 2/6 from roasted peanut (R-Ara h 2/6) had a similar albeit slightly reduced immunoreactivity to N-Ara h 2/6.

**Table 1:** Serological and clinical information for the 34 peanut allergic patients recruited in different European centres, within the Europrevall European project, used in EAST studies, basophil histamine release tests and/or PBMC proliferation tests.

Code	Gender (f/m)	Age	Peanut SPT (HEP index numbers)	Grade peanut allergy	Peanut RAST class	peanut CAP (IU/ml)	Anti-whole peanut IgE (IU/ml)	Anti-Ara h 2/6 IgE (IU/ml)	Other food allergies
2A	m	31	positive	3/4	5	51.7	43	31	
52A	m	41	nd	4	5	nd	166	62	
54A	f	11	2.2	1	6	>100	72	57	
55A	f	14	3.3	4	6	>100	363	137	
56A	m	16	1.5	2	6	>100	79	56	
58A	f	22	2.5	4	6	>100	171	110	Soy, lupine, pea
65	f	19	positive	3/4	6	>100	587	208	
66	m	18	positive	4	6	>100	290	71	
67	f	46	nd	3	5	54.4	95	35	Hazelnut, kiwi, cherries
68	f	20	positive	1	4	17.6	16	6	
69	m	21	positive	3/4	3	6.98	3.5	1.2	
70	f	21	positive	3/4	6	>100	224	76	Soy, pea, apple
73	f	22	positive	2/3	4	33.6	21	3.7	Hazelnut
76	m	23	positive	3	3	8.6	4.2	0.3	
77	m	25	positive	2	4	33.2	22	3.5	
78	f	24	positive	3	5	79.6	65	29	Hazelnut
80	m	27	positive	3	3	16	12	17	
82	f	18	positive	1/2	3	4.7	25	6.4	Kiwi, cherries
1682	m	33	nd	4	6	>100	13	10	
1691	m	22	nd	3	5	nd	50	54	
2207	m	28	nd	4	4	nd	38	26	Wheat, hazelnut, egg, almond
2209	f	27	nd	4	6	nd	75	51	Hazelnut, egg, wheat
2304	m	25	nd	3	6	nd	90	50	
2305	f	18	nd	4	nd	nd	52	36	
03–0043	f	20	1,54	3/4	6	>100	nd	852	Hazelnut, walnut, melon
03–0044	m	23	4,33	3	6	>100	nd	231	Egg, fish, shrimp, hazelnut, walnut, sesame, wheat, sunflower
03–0079	f	16	2,22	3	6	>100	nd	88	Pistachio, almond, brazil nut
05–0128	f	25	1,92	3	6	>100	nd	358	Soy, hazelnut, apple, peach
05–0165	f	18	0	3/4	6	>100	nd	429	Chickpea, bean, hazelnut, lentil
05–0195	m	31	2,13	3	5	63,5	nd	64	Soy
05–0209	m	20	99,98	4	5	70,2	nd	81	Pea, soy, bean
06–0010	m	19	2,2	3/4	5	67,4	nd	67	Fish
08–0069	m	17	2,08	1	6	>100	nd	139	Kiwi
12–0048	f	21	5,8	3/4	5	54,6	nd	88	Shrimp

HEP: histamine equivalent prick test; nd: not determined. Where known, other allergies of the subjects are indicated.

The origin of the sera is as follows: 2A: Zurich, 52A: Amsterdam, 54A-82: Arnhem, 1682-2305: Vienna; 03-0043 till 12-0048 EuroPrevall Serum Bank.

Grading is based on Brockow and Ring and on Sampson *et al.* [41], [42]. Grade 1 = dermal symptoms; grade 2 = gastro-intestinal problems like nausea and or cramping; grade 3 = any of the former grades plus vomiting/diarrhoea and respiratory tract problems like throat pruritis or tightness, grade 4 = any of the former grades and respiratory arrest plus cardiovascular problems like hypotension.

### Effect of thermal processing on the mediator-releasing capacity

**Figure 5:**
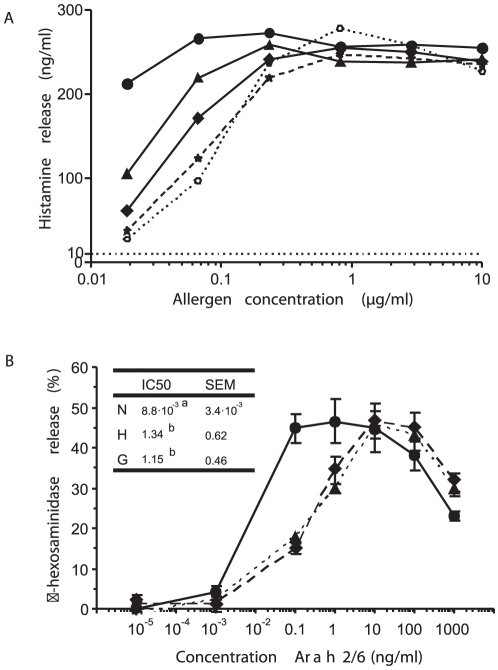
Effect of thermal treatment on the activation of effector cells induced by Ara h 2/6. (**A**) Human stripped basophils were passively sensitized with individual sera (#2305) and then incubated with increasing concentrations of N-Ara h 2/6 (•), H-Ara h 2/6 (⧫), and G-Ara h 2/6 (▴), or whole peanut extract from raw (★) or roasted (○) peanut. Histamine release was assayed in corresponding supernatants. Twenty-three sera out of the 35 available corresponding to all sera from Zurich, Amsterdam, Vienna and EPSB, and sera 54A to 58A from Arnhem were used, giving similar results (not shown). No histamine release was induced by the different allergens when using a serum from patients not sensitized to peanut (data not shown). (**B**) Humanized RBL-2H3 cells were passively sensitized with sera from peanut-allergic patients (#70) and stimulated with increasing concentrations of native (N; ―•―), heated (H; – –⧫– –) or glycated (G; - - -▴- - -) Ara h 2/6. Error bars represent the SD of triplicate values. No β-hexosaminidase release was induced by the different allergens when using sera from non-allergic patients (data not shown). The table presented within the figure represents the average protein concentrations (ng/ml, n = 6) to obtain 50% of the maximum allergen release of the native allergen (EC50). Means without a common letter differ (P<0.05).

Subsequently an analysis of the effects of heating on the histamine releasing capacity of the Ara h 2/6 preparations was undertaken using human stripped basophils passively sensitized with 23 of the 34 peanut-allergic patients sera and compared with whole protein extract from raw or roasted peanut. No histamine release was induced by the different allergens when using a serum from patients not sensitized to peanut (data not shown), and the 23 sera from PA patients gave similar results. An example curve (serum #2305, Fig. 5A) shows significant histamine release for both extracts and purified allergens, the whole protein extract from raw and roasted peanut being comparable. As previously observed using RBL SX38 cells and Ara h 2 and Ara h 6 from roasted peanut [22], histamine release was induced at lower concentrations of these purified allergens when compared to peanut extracts. Heating reduced the biological potency of the Ara h 2/6, (Fig. 5A, P<0.05 when considering 23 sera), but intriguingly histamine release was induced at a lower concentration of heated-glycated than heated Ara h 2/6. The maximum histamine release induced by the purified allergens was only affected when comparing N-Ara h 2/6 to H-Ara h 2/6 (P<0.05 when considering the 23 sera).

Additionally, a mediator release assay using humanized Rat Basophilic Leukaemia cells was also performed using 6 individual sera. Figure 5B shows typical results of a representative serum (#70) and the inserted table represents the average protein concentrations needed to obtain 50% of the maximum mediator release induced by the native allergen (EC50). A 150 and 130 times increased EC50 value, corresponding to a decreased allergenic activity, was observed for H-Ara h 2/6 and G-Ara h 2/6, respectively, in comparison to N-Ara h 2/6. The EC50 value of G-Ara h 2/6 was lower compared to the EC50 value of H-Ara h 2/6; however, this was not tested to be significantly different.

## Discussion

Boiled peanuts are consumed in many countries, from China to the Southern states of the USA. Boiling is performed for 2 to 7 hours, depending on the peanut source, and boiled peanut can be further canned, then involving further extensive heating (45 minutes at 120°C under 10 pounds pressure per square inch). Study of heat-induced structural changes and its impact on allergenicity of peanut allergens after wet-thermal processing then appears of interest. Achieving model processing conditions to ensure that thermal modifications can be monitored by structural and immunological analysis is difficult since heating frequently renders much of the protein insoluble. This makes purification of proteins from cooked foods, such as roasted or boiled peanuts, cumbersome [23]. We then analyzed the effect of wet thermal processing on major peanut allergens, i.e. N-Ara h 2/6, previously purified from raw peanut. Like other members of the prolamin superfamily, the 2S albumin allergens of peanut appeared to be highly thermostable. The present detailed molecular study shows that these proteins only begin to unfold following heating at temperatures over 100°C, conditions which equate to boiling for extended times (longer than 15 min). After this time the protein adopts a random coil conformation and forms dimers and higher order oligomers, accompanied by hydrolysis of the peptide backbone. Such effects of thermal denaturation have been observed for other food proteins, including the lipid transfer protein from barley which shares the same protein scaffold as the 2S albumins [24]. Interestingly, the Ara h 2 and 6 purified from roasted peanuts had retained the structural characteristics of the native protein purified from raw peanuts, as indicated by far-UV CD spectra and non-denaturing electrophoresis. In addition, proteins from roasted peanut have a similar IgE immunoreactivity as the unheated protein. These data thus demonstrate that the proportion of the protein in roasted peanut that was still soluble and then extractable had not been denatured by the roasting process, probably due to protection within the peanut seed.

In order to assess the effect of heat-induced structural changes on allergenicity of Ara h 2/6, the *in vitro* proliferation and cytokine production capacity of the native *vs* heated proteins were tested using PBMC from 12 peanut allergic (PA) and 12 control human donors. Out of the 12 PA, only 3 demonstrated significant proliferation, as assessed by Ki-67 labeling, and 5 demonstrated a significant cytokine production after *in vitro* stimulation. All of them had high levels of peanut specific IgE antibodies. This corroborates studies demonstrating a significant positive correlation between allergen-specific serum IgE levels and high numbers of IL-4-producing cells [25] or active proliferation of T cells and Th2 cytokine production [26]. However, it is worth noting that peanut-specific T-helper cells in PBMC of peanut allergic patients were found to be only 0.6% of the total CD4+ T cells [27], thus explaining the quite low rate of responders in our *ex vivo* stimulation assays. However this does not preclude that heat treatment of Ara h 2/6 did not influence cytokine production by PBMC from all of the 5 PA responders, regardless of the presence of glucose. Interestingly, internalization of glycated ovalbumin was recently studied in human dendritic cells and an increased uptake of glycated ovalbumin compared to the native protein was observed, which finally led to increased CD4+ T-cell immunogenicity of this protein [14], [15]. However, in our study, monocytes are the primary antigen-presenting cell population, T cells are already primed by the proteins, and the used model allergens are different, which renders comparison difficult. Our results demonstrated that the hydrolysis, unfolding and aggregation of the Ara h 2/6 by intense heating, regardless of the presence of glucose, had no effect on stimulating the activity of PBMCs from PA-patients, as has been shown before for Bet v 1-related allergens [28], [29]. This demonstrated that T-cell epitopes were still present in the modified proteins.

The IgE-binding capacity and the elicitation potency of the native *vs* heat-processed allergens were then studied using a large panel of sera from peanut allergic patients (n = 30 and 23, respectively). In fact, most of the studies aiming at analyzing the relationship between structure and allergenicity, and particularly the effect of processing on allergenicity of food proteins, generally use very few individual sera from allergic patients [18], [23], [30], [31], even sometimes only one pool of such sera is available [11], [19], [32], [33]. In the present study, a large population of peanut allergic patients from various European countries was involved leading to significant conclusions. Many studies have demonstrated that physicochemical modifications of proteins, e.g. resulting from processing, may impact on their IgE binding capacity. This includes denaturation or hydrolysis which may lead to the destruction of conformational epitopes, and thus to a decreased IgE binding capacity. Inversely, structural modifications may result in the unmasking of epitopes hidden in the tertiary structure of the molecule and that thus become available for IgE binding. The European Food Safety Authority opinion relating to the evaluation of allergenic foods for labelling purposes also acknowledged that the data available do not indicate whether and how food processing predictably influences allergenicity [34], [35]. With regards to peanut 2S albumins, several linear epitopes have been identified for Ara h 2 [36], [37], [38], [39] and some of them have been proposed as a marker of persistent peanut allergy [38]. However, the relative binding of IgE to the corresponding peptides *versus* to the native protein was not analyzed. Interestingly, Bernard and coworkers showed that the IgE binding capacity of Ara h 6 was decreased for all the peanut allergic patients in their study and even abolished for 25% of them when the molecule was denatured by reduction and carboxymethylation. This demonstrates the importance of conformation in the allergenicity of this protein and the presence of both conformational and linear epitopes, at least for 75% of their population [6]. In the present study, heat-induced denaturation, hydrolysis and aggregation of Ara h 2/6 result in a significant loss in IgE-binding capacity and ability of the protein to elicit histamine release for all tested sera. Altogether, these results suggest that IgE responses in peanut-allergic individuals are mainly directed towards the natively folded protein. Hydrolysis and oligomerisation observed after heating with or without glucose, may have led to further linear epitope degradation and masking. Thus, it appears that the sensitizing agent driving B-cell responses in this population was mainly native Ara h 2/6 which may have originated from either consumption of raw nuts, or more probably, represent the fraction of Ara h 2/6 that remains soluble and in its native state even after roasting. However, this also indicates that thermal processing can reduce the allergenic activity of peanut proteins, and may explain why processes such as boiling of peanuts, can reduce their allergenic activity [32].

This study showed that glycation in conjunction with thermal denaturation generally led to a further decrease of IgE binding capacity, rather than the increase found by others [18], [40], although glycation did appear to preserve slightly more of the proteins mediator releasing capacity compared with heating alone. Some of these differences may relate to the wet-thermal processing procedures employed in this study. Further investigation using heat-denaturation of Ara h 2/6 under low moisture conditions will be required to explain these differences more fully.

In conclusion, heating to temperatures able to denature Ara h 2/6 caused a significant decrease in the proteins allergenicity whereas T cell stimulation was not affected which can render these modified proteins attractive for immunotherapy. It is evident that a soluble fraction of the Ara h 2/6 from roasted peanuts retains the conformation and allergenic activity of the native protein, explaining the allergenic potency of this protein.

## Materials and Methods

### Ethics statement

A written informed consent was obtained before the sample collection (serum or serum and PBMC) and the performed experiments were approved by the corresponding local ethical committees (Kantonale Ethikkommission Zürich, Medical ethical committee of the Amsterdam Medical Centre, Ethics Committee of Medical University of Vienna, Commissie Mensgebonden Onderzoek regio Arnhem-Nijmegen).

### Patient characteristics

Thirty-four peanut-allergic (PA) patients (17 males and 17 females, mean age: 23 years) were recruited in Zurich, Amsterdam, Vienna and Arnhem, or were provided by the Europrevall Serum Bank (EPSB) (table 1). Peanut allergy was established using clinical history, physical examination, peanut specific IgE (ImmunoCAP and/or RAST (Phadia AB)) and/or objective clinical manifestations observed after peanut ingestion. Grading of food-induced anaphylaxis was according to severity of clinical symptoms [41], [42]. Twelve PA subjects and 12 non-allergic (NA) controls, all recruited from the Allergology Practice Arnhem (APA, The Netherlands), donated cells for PBMC cultures.

### Peanut 2S albumins (Ara h 2/6) preparations

Native 2S albumins containing both Ara h 2 and Ara h 6 (N-Ara h 2/6) were purified from unroasted redskin type peanut as described previously [21] and stored at −20°C prior to use. This purification involves ammonium sulphate fractionation and gel-filtration chromatography, avoiding the use of denaturing conditions such as reverse phase HPLC. Allergen identity was confirmed by in gel-trypsin digestion and MALDI mass spectrometry. Ara h 2 and Ara h 6 were purified from commercial roasted peanut as previously described [20] which involves anion exchange chromatography in 4 M urea followed by reverse phase HPLC, and then mixed (62% Ara h 2 and 38% Ara h 6) in proportions representative of proportions found in peanut [43] to provide a preparation further referred to as R-Ara h 2/6.

Proteins were standardized according to BCA kit from Pierce, following provider recommendations.

### Heating and glycation treatments of peanut 2S albumins (Ara h 2/6)

Native Ara h 2/6 (N-Ara h 2/6, 4 mg/ml) in 32.5 mM phosphate buffer containing 100 mM NaCl, was heated for 15 min to 110°C alone (H-Ara h 2/6) or in the presence of 100 mM glucose (G-Ara h 2/6). Heating in the presence of glucose was undertaken to glycate the protein based on a previously used protocol [44]. Protein solutions were allowed to cool to room temperature prior to analysis.

### Biochemical characterization of native and modified Ara h 2/6

Changes in Ara h 2/6 secondary structure were assessed using far-UV (190–260 nm) circular dichroism (CD) spectroscopy using either a J-710 or a JASCO-810 spectropolarimeter (Jasco Ltd., Japan) as previously described [7], [21]. All data were calculated in terms of molar ellipticity. Aggregation state was monitored using size exclusion chromatography on a Superdex-75 column attached to an Äkta Basic FPLC (Amersham Biosciences, Little Chalfont, UK) [21], and calibrated with a set of gel filtration molecular weight standards (BioRad, Hertfordshire, UK).

### Isolation, culture and stimulation of human peripheral blood mononuclear cells

PBMC were isolated and cultured as previously described [45]. Immunological phenotyping of freshly isolated PBMC was performed on a FACS Canto II (BD Pharmingen, San Diego, USA), using monoclonal antibodies and the procedure from BD Pharmingen (San Diego, USA). Stimuli were incubated for at least 30 min with polymyxin B (50 μg/ml, Sigma, Zwijndrecht, The Netherlands) to remove potential endotoxin activity prior to addition of the stimuli to the PBMC. PBMC were stimulated with αCD3/αCD28 (150 ng/ml αCD3, 100 ng/ml αCD28) or N-, H- or G-Ara h 2/6 (20 μg/ml) and supernatants harvested after 4 and 7 days of culture and stored at −80°C. The 20 µg/ml dose was determined as optimal in preliminary studies using doses ranging from 5 to 40 µg/ml (data not shown). The specificity of the proliferative and cytokine production capacities were assessed in a pilot study using PBMC from 5 peanut allergic patients with 2 control proteins (ovalbumin and purified 7S globulin from soy (Gly m 5), 20 µg/ml). No differences between the medium control and the control proteins with respect to cytokine induction and proliferation were observed. Early apoptosis and late apoptosis/necrosis was assessed on freshly prepared and 7 day cultured cells using double staining with APC-Annexin V and propidium iodide (PI) [46]. PBMC proliferation was studied on day 4 and day 7 by analysis of intracellular expression of the nuclear Ki-67 antigen (BD Pharmingen, San Diego, USA) [46] of PBMC previously stained with anti-CD4/CD25 antibody. Ki-67 expression by CD4^+^ and CD25^+^ cells was then determined by flow cytometry.

### Cytokine analysis

Cytokine production by PBMC was analysed in supernatants of cells cultured for 4 and 7 days. The production of IL-5, IL-10, IL-13, IFN-γ and IL-17 by PBMC cultures was determined in harvested supernatants using Cytometric Bead Array (CBA, BD Biosciences, San Diego, CA) on a FACSCanto II cytometer and the procedure was performed according to the manufacturer's protocol. Preliminary experiments demonstrated low specific induction of IL-4 that correlated to higher specific induction of IL-5 and IL-13, all indicative of Th2 cellular responses. Therefore, IL-4 was not determined in the full experiments using PBMC from 12 NA and 12 PA donors. The detection limit was 1.1 pg/ml for IL-5, 0.13 pg/ml for IL-10, 0.6 pg/ml for IL-13, 1.8 pg/ml for IFN-γ and 0.3 pg/ml for IL-17.

### IgE immunoreactivity

Concentrations of IgE specific to whole protein extract from raw peanut and to N-Ara h 2/6 were determined using enzyme allergosorbent tests (EAST) as previously described [3], [20].

Reverse EAST inhibition was performed as described elsewhere [47] using anti-human IgE (mouse monoclonal, clone LE27) as a capture antibody, biotinylated N-Ara h 2/6 (200 ng/ml) as tracer and N-, H-, G- or R-Ara h 2/6 as inhibitors. N-Ara h 2/6 was labelled with biotin using EZ-link sulfo-NHS-LC biotin (Pierce, Rockford, IL). Assays were developed using streptavidin labelled with acetylcholine esterase (AChE) and Ellman's reagent. The absorbance of each well was measured at 414 nm and results expressed as *B*/*B*0, where *B*0 and *B* represent the amount of native Ara h 2/6 tracer bound to captured IgE in the absence or presence of a known concentration of inhibitor, respectively.

### Effector cells activation by natural and modified allergens

Basophil histamine release (BHR) tests were performed as described in [49] using stripped basophils from fresh buffy coats (Blood Bank, National University Hospital of Copenhagen, Denmark) and reagents from RefLab (Copenhagen, Denmark). Twenty-three sera out of the 34 available were used individually in the BHR test, corresponding to all sera from Zurich, Amsterdam, Vienna and EPSB, and sera 54A to 58A from Wageningen.

RBL-2H3 cells expressing the α-chain of the human FcεRI receptor [49] were kindly provided by Drs. Vieths and Vogel (Paul-Ehrlich-Institut, Langen, Germany). RBL cells were cultured in MEM medium supplemented with 5% fetal calf serum and 1% glutamine (all from Gibco-BRL, Paisley, UK) at 37°C in a humidified atmosphere with 5% CO_2_. Cells in stationary growth phase were harvested and plated in 96-well plates at 1.5×10^5^ cell/well. Six sera from PA patients (#65, #66, #67, #70, #55A and #56A) at pre-determined convenient dilutions and two sera from non-allergic patients were added and incubated overnight to passively sensitize the cells. After washing, the cells were stimulated for 1 h with the allergens diluted in Tyrode's buffer containing 50% deuterium oxide [49]. The antigen-specific release was quantified by measuring β-hexosaminidase activity and expressed as percentage of the total β-hexosaminidase content that was obtained by lysing the cells with Triton-X100 (Sigma-Aldrich, Zwijndrecht, The Netherlands). The release data were fitted to four-parameter-logistic curves by non-linear regression using the SigmaPlot 10.0 software package, and EC50 values were calculated accordingly.

### Statistical analysis

Statistical analysis was performed by using SPSS (v18.0, SPSS Inc., Chicago, USA) or GraphPad Prism v4.00 for Windows (GraphPad Software, San Diego, CA). Means and medians were analysed using Wilcoxon signed rank test for specific IgE levels and PBMC cytokine production and the Mann-Witney U test for comparing PBMC cytokine production between PA and the control patient groups. Differences were interpreted as significant when P<0.05.
